# Encapsulation of Iron-Saturated Lactoferrin for Proteolysis Protection with Preserving Iron Coordination and Sustained Release

**DOI:** 10.3390/nano13182524

**Published:** 2023-09-08

**Authors:** Przemysław Gajda-Morszewski, Anna Poznańska, Cristina Yus, Manuel Arruebo, Małgorzata Brindell

**Affiliations:** 1 Department of Inorganic Chemistry, Faculty of Chemistry, Jagiellonian University in Kraków, Gronostajowa 2, 30-387 Krakow, Poland; pgmorszewski@doctoral.uj.edu.pl (P.G.-M.); anna4.poznanska@student.uj.edu.pl (A.P.); 2 Doctoral School of Exact and Natural Sciences, Jagiellonian University in Kraków, Prof. St. Łojasiewicza St 11, 30-348 Krakow, Poland; 3 Instituto de Nanociencia y Materiales de Aragón (INMA), CSIC-Universidad de Zaragoza, 50009 Zaragoza, Spain; cyargon@gmail.com; 4 Department of Chemical Engineering, University of Zaragoza, Campus Río Ebro-Edificio I+D, C/Poeta Mariano Esquillor S/N, 50018 Zaragoza, Spain

**Keywords:** lactoferrin, protein encapsulation, protein delivery, microparticles

## Abstract

Lactoferrin (Lf) is a globular glycoprotein found mainly in milk. It has a very high affinity for iron(III) ions, and its fully saturated form is called holoLf. The antimicrobial, antiviral, anticancer, and immunomodulatory properties of Lf have been studied extensively for the past two decades. However, to demonstrate therapeutic benefits, Lf has to be efficiently delivered to the intestinal tract in its structurally intact form. This work aimed to optimize the encapsulation of holoLf in a system based on the versatile Eudragit^®^ RS polymer to protect Lf against the proteolytic environment of the stomach. Microparticles (MPs) with entrapped holoLf were obtained with satisfactory entrapment efficiency (90–95%), high loading capacity (9.7%), and suitable morphology (spherical without cracks or pores). Detailed studies of the Lf release from the MPs under conditions that included simulated gastric or intestinal fluids, prepared according to the 10th edition of the *European Pharmacopeia*, showed that MPs partially protected holoLf against enzymatic digestion and ionic iron release. The preincubation of MPs loaded with holoLf under conditions simulating the stomach environment resulted in the release of 40% of Lf from the MPs. The protein released was saturated with iron ions at 33%, was structurally intact, and its iron scavenging properties were preserved.

## 1. Introduction

Lactoferrin (Lf), a protein commonly found in fluids and mammalian secretions, has attracted the attention of many researchers due to its numerous biological activities. The antimicrobial, antiviral, anticancer, and immunomodulatory properties of this protein have been widely discussed and included in recent reviews [1,2,3,4,5]. Lf (78–80 kDa) has two homologous lobes, each of which can coordinate one metal ion, as confirmed by its crystal structures [6,7]. Native Lf is partially saturated with ionic iron because of its very high affinity for these ions (K_a_~10^22^–10^23^ M^−1^) [8]. Lf was shown to exhibit its biological activity depending on the level of metal ion saturation [8,9]. Fe-depleted Lf (apoLf) was reported to exhibit antifungal and bacteriostatic activity, whereas the Fe^3+^-saturated protein (holoLf) displayed immunomodulatory and anticancer activity [9,10,11]. Lf itself was also reported to eliminate bacterial pathogens using an iron-independent mechanism [9]. 

As a result of its multipotential activity, Lf is a biologically relevant molecule that is the subject of numerous studies. Researchers show interest in designing new therapies in which molecules of natural origin, such as Lf, are used to induce a synergistic effect with antimicrobial, antiviral, and anticancer drugs [12,13]. In our recent mini-review, we discussed the potential of Lf as a synergistic agent and outlined the suggested mechanisms that underlie its observed activity [11]. Particular attention was paid to research that demonstrated the adjuvant properties of holoLf in anticancer chemotherapy. In vivo studies conducted on mice by Kanwar et al. [14] and Sun et al. [15], among others, revealed that the combination therapy of holoLf and anticancer drugs was significantly more effective than the chemotherapy alone with limited side effects. Furthermore, mice fed with a holoLf-enriched diet were able to overcome tumor resistance during chemotherapy [14]. A high level of saturation of Lf with ionic iron was essential to achieve adjuvant properties in anticancer treatment [14,15]. Furthermore, supplementation with bovine Lf (native, partially saturated with iron ions) showed therapeutic benefits in reducing side effects during antibiotic therapy [16] or chemotherapy [17,18]. In in vivo studies, lactoferrin exhibited modulatory and normalizing properties in mice with dysbiosis induced by antibiotics [16]. Similar normalizing activity was shown in patients with colorectal cancer receiving chemotherapy in a clinical trial [18]. The symptoms of cancer in patients with metastatic colorectal cancer (e.g., anemia) eased, and side effects decreased (the kidney and liver were protected from drug toxicity, and related clinical parameters were improved) [18]. The authors claimed a significant therapeutic effect of Lf if used for a long time; however, long-term cohort studies are still required to corroborate this finding [18]. The research carried out so far shows that the use of Lf, especially the iron-saturated one, might have a beneficial effect on cancer treatment. In general, lactoferrin can be considered functional food, and according to the European Food Safety Authority (EFSA), it is safe to use in foods and food supplements (EU Commission Decision No 2012/727/EU [19]).

Although Lf in the apo form or saturated with iron ions is very promising as an adjuvant agent in antimicrobial or anticancer treatment, there are a few obstacles that must be overcome by its appropriate delivery to the site of action. Without a doubt, oral administration should be considered as the most convenient drug delivery option, especially in the case of diseases that affect the gastric tract. However, Lf undergoes proteolysis in the stomach, like many other proteins. The digestion of Lf in vivo is poorly understood, and there are a lot of contradictory claims about conditions that should be applied to obtain a reliable model. Thus, the exact mechanisms of proteolysis are unknown as physicochemical conditions in the human digestive system are hard to mimic [20]. Currently, most researchers indicate that Lf cannot pass through the proteolytic environment of the stomach in its structurally intact form based on in vitro [21,22,23,24,25] and in vivo [26,27,28,29] studies. Studies on mice show that digestion of lactoferrin is almost completed within 2 h [29]. However, the results strongly depend on the applied conditions such as pH, digestion time, or gastric emptying time. On the contrary, some researchers imply that 60–99% of Lf can pass through the stomach in the structurally intact form [30]. In the case of newborns, the digestive system of infants is not mature enough, so intestinal permeability is enhanced [31], and the optimal pH for the action of digestive enzymes is not reached. Most of the Lf can pass in an intact form through the digestive system under those conditions [32]. Furthermore, even if Lf passes through the stomach in a structurally intact form, it loses its coordinated Fe^3+^ ions, which might be crucial for its biological activity [33]. In an acidic environment, the ligands that coordinate Fe^3+^ undergo protonation; as a result, the binding sites are destabilized, and iron ions are released. 

To address the mentioned problems and ensure that iron-saturated Lf is effectively applied, an adequate delivery system to promote its use alone or in combination therapies with other drugs needs to be designed. A broad diversity of systems for protein encapsulation and delivery was proposed in the literature. Diverse solutions are available for sustained or immediate release and for targeted delivery, depending on the intended use. The achievements in the field to date are summarized in the latest reviews [7,34,35]. However, despite numerous methods, most available systems have low encapsulation efficiency (usually around 50–75% [7,34,36,37,38]), which may make them unprofitable. In this study, a system based on the versatile Eudragit^®^ RS polymers, previously used for the extremely efficient encapsulation of bovine serum albumin (EE% 88.4%) and horseradish peroxidase (EE% 95.8%) by Mendoza et al. [39], has been optimized and tailored to protect holoLf from gastric fluid degradation. The chosen polymer belongs to a family of methacrylate copolymers with the commercial name Eudragit^®^. This basic methacrylate copolymer was generally recognized as safe (GRAS) by the FDA (GRAS Notice (GRN) No. 710 [40]) and was approved as a food additive in the EU (under the number E 1205; EU Commission Regulation No 1129/2011 [41]). The side chains of Eudragit^®^ may be modified, providing whole families of polymers with different properties. Wise tailoring properties allow the polymer to be well-fitted for the purpose (pH-dependent or pH-independent polymer for sustained or immediate release) [42]. Eudragit^®^ has already been used in many pharmaceutical formulations [43]. It is known to be inert and biocompatible. Its fate upon oral administration was studied in numerous reports summarized by Eisele et al. in their paper prepared for the purpose of Eudragit GRAS evaluation and designation [44]. Basic methacrylate copolymers are excreted through the feces in their unchanged form, with a mean total of 93.3% within 48 h. Minor absorption is possible but on a very low level, which is less than 0.02% of the administered dose [40,44]. Additionally, there are limited data about systems designed for the oral delivery of iron-saturated lactoferrin protecting from iron ion dissociation in the acidic pH of the stomach [45]. The alginate-enclosed chitosan–calcium–phosphate nanocarrier proposed by Kanwar et al. was the first and only system (to the best of our knowledge) that was designed to keep lactoferrin saturated with iron ions [46].

In this paper, we propose an entirely new fast-prepared encapsulation system based on Eudragit^®^ RS. The release of the entrapped protein from the MPs is reported, and the characterization of the released lactoferrin is focused on evaluating its resistance to proteolysis, and the preservation of its iron saturation level, structure, and ionic iron scavenging potential.

## 2. Materials and Methods

Eudragit^®^ RS was kindly donated by Evonik Industries AG (Essen, Germany), bovine Lf-NFQ^®^ (Lf purity > 95%) purchased from Taradon Laboratory (Tubize, Belgium) was saturated with Fe^3+^ ions as previously described [47], and the obtained iron saturation level was 98.3% as determined using a mass spectrometry with inductively coupled plasma (ICP-MS) method described in Section 2.1. Polyvinyl alcohol (PVA) (MW 89,000–98,000 g/mol; 99+% hydrolyzed) was purchased from Sigma-Aldrich (Saint Louis, MO, USA). All other chemicals were purchased from Sigma-Aldrich or Avantor (Gliwice, Poland) and were at least analytical grade; the solvents for HPLC were purchased from Sigma-Aldrich and were gradient grade. MiliQ class water (Merck Millipore system, Darmstadt, Germany) was used to prepare all the aqueous solutions. Protein concentration was determined via the bicinchoninic acid (BCA) assay using the Pierce BCA Protein Assay Kit (23225, Thermo Fisher Scientific, MA, USA) according to the manufacturer’s protocol. Potential interferences between the Eudragit^®^ RS system and the protein determination were excluded (see the Supplementary Materials section).

### 2.1. Iron Saturation Determination 

ICP-MS was used to monitor the Lf iron saturation level. To eliminate interferences, a collision–reaction cell system was used with the kinetic energy discrimination (KED) method using helium as an inert gas. The samples were mineralized in concentrated nitric acid (Sigma-Aldrich, trace metal basis, ≥99.999%) for 24 h at 60 °C, diluted in MiliQ class water, and analyzed using ICP-MS spectrometer (NexION 2000C, Perkin Elmer, Waltham, MA, USA). Protein concentration was determined using BCA and HPLC chromatography, as described in Section 2.3 and Section 2.4, respectively. It must be noted that the iron saturation level of Lf was defined as the percentage of iron-binding sites occupied by ferric ions, assuming that 2 mols of Fe^3+^ ions were bound per 1 mol of protein.

### 2.2. Protein Encapsulation 

The water-in-oil-in-water double emulsion method (W/O/W) using solvent evaporation developed by Mendoza et al. [39] was optimized for holoLf encapsulation into Eudragit^®^ RS microparticles (MPs). The aqueous phase contained 10 mg of holoLf in 1 mL of MiliQ water. The aqueous phase was sonicated with an organic phase containing 100 mg of Eudragit ^®^ RS dissolved in 2.5 mL of CH_2_Cl_2_, with one drop of triethyl citrate (Avantor) to obtain the first emulsion. Sonication was carried out for 35 s with an amplitude of 35% on a QSonica 500 (Newton, CT, USA) equipped with a 1/8″ probe. Subsequently, the first emulsion was immediately sonicated with 2 mL of 1% (*w*/*v*) PVA in an acidic solution (pH~4.5, acetic acid) to obtain the final W/O/W emulsion. An additional 10 mL of 0.3% (*w*/*v*) PVA acidic solution was added to ensure stability and the emulsion was stirred at 600 rpm for 3 h to evaporate the dichloromethane. The MPs were washed three times with acidic water (pH~4.5 acetic acid) and collected via decantation. MPs were used for further experiments immediately after collection, or frozen in liquid nitrogen, and then lyophilized. The freeze-drying process was performed using an Alpha 1–2 LD plus lyophilizer (Christ, Germany) for 18 h under vacuum 0.31 bar, and the condenser temperature was set to −32 °C. One mL of MiliQ water was used as the aqueous phase to obtain empty MPs for comparison.

### 2.3. Microparticles Characterization 

Lyophilized MPs were used for characterization. The synthesis yield was determined as the ratio between the mass of the obtained MPs after lyophilization and the mass of all the chemicals used during the synthesis. Scanning electron microscopy (SEM) performed on the Tescan VEGA 3 microscope (Tescan Orsau Holding a.s., Brno, Czech Republic) provided images for morphological studies and the determination of the particle diameter distribution. MPs were coated with a thin layer of gold (Quorum Q150R coater) prior to SEM imaging. SEM images were analyzed using ImageJ 1.53e [48,49] to determine the diameter distribution, and at least 100 objects from each MP sample were imaged to ensure significance. The entrapment efficiency (EE%-ratio between the mass of the entrapped protein and the mass of total protein used for the encapsulation) and the loading capacity (LC%-ratio between the mass of the entrapped protein and the overall mass of the MPs) were determined by quantifying the holoLf with the BCA assay. The EE% was determined in two ways: directly and indirectly. In the direct approach, the lyophilized MPs were suspended in water, sonicated until the disintegration of the MPs (pulse sonication, 10 cycles of 10 s at 40% amplitude), and centrifuged (15 min 14,000× *g*). The released protein was quantified in the supernatant. The indirect approach was based on the quantification of holoLf using the BCA assay in the supernatant above pelleted MPs after evaporation of the organic phase at the end of the MP synthesis. Entrapped protein was calculated using a mass balance as the difference between the total protein used for encapsulation and the protein remaining in the solution.

### 2.4. Protein Release from the MPs 

The release profile was investigated in 10× diluted PBS with MPs after lyophilization, as well as with freshly prepared MPs that were used in suspension immediately after encapsulation. MPs were incubated at room temperature under stirring at 300 rpm. The release profile was studied for 24 h. At defined time points, aliquots of the suspension were taken and centrifuged (10 min 5000 rpm), and the released protein concentration was determined using BCA. The percentage of the released protein was calculated as the mass of the released protein relative to the total mass of the protein in the MPs (calculated from the mass of MPs and LC%).

Furthermore, the release of Lf from the MPs was studied in simulated gastric fluid without enzymes (GF, 34.2 mM NaCl, 0.08 M HCl, pH~1.1) or using a proteolytic enzyme (GF + E: GF enriched with 1 mg/mL pepsin; P7125, Sigma-Aldrich, >400 units/mg protein), and simulated intestinal fluid (IF, 15.4 mM NaOH, 50 mM KH_2_PO_4_, pH~6.8) prepared according to the 10th edition of the *European Pharmacopeia*. For the evaluation of the Lf release, freshly prepared MPs were used in suspension immediately after synthesis. MPs were washed three times with GF (or GF + E) and suspended in a fresh portion of GF (or GF + E) under sink conditions and incubated at room temperature under stirring at 300 rpm for 1 h. The supernatant was collected after 1 h and the remaining MPs were washed three times with IF, suspended in a fresh portion of IF, and incubated under stirring for another 23 h. After 1, 3, and 23 h aliquots of supernatant were collected for further analysis. An additional sample of MPs was suspended in IF, and the aliquots of supernatant were collected after 1, 2, 4, and 24 h as a reference to monitor total release without protein degradation (pH- or enzyme-dependent). Furthermore, the remaining MPs were collected after each experiment and subjected to sonication (pulse sonication, 10 cycles of 10 s at 40% amplitude) to achieve a full disintegration of the MPs and full protein release. All samples were centrifuged (14,000× *g* for 30 min), and the collected supernatants were subjected to HPLC separation to evaluate the Lf content and stability. Chromatographic separation was performed on a Shimadzu LC-2030c 3D plus system with a Brownlee BioC18 column (particle size 5 μm; dimension 150 × 4.6 mm; Perkin Elmer). Mobile phase A was 0.05% Trifluoroacetic acid (TFA) in water. Mobile phase B was acetonitrile enriched with 0.05% TFA. A gradient elution from 20% B was applied to 70% B over 30 min with a flow rate of 1 mL/min, and the temperature of the column compartment was maintained at 30 °C. The detection wavelength was set to 280 nm and the injection volume was 20 μL. Solutions of Taradon Lf-NFQ^®^ with concentrations of 5, 10, 17.5, and 25 μM were used as standards for checking HPLC separation in terms of linearity and stability of the column (Figures S1 and S2). Additionally, the released Lf was examined for any structural changes using circular dichroism (CD), and the iron saturation level and the ability to rebind iron ions were examined using mass spectrometry (ICP-MS), as described in infra.

### 2.5. Protein Stability and Functionality Studies 

The stability of the released Lf was evaluated based on CD spectra and HPLC chromatograms (the separation parameters are described in Section 2.4). All CD spectra were recorded on a Jasco J-815 spectropolarimeter, with a standard-sized sample compartment and a cuvette with a 1.00 mm path length, under a nitrogen flow rate of 4 L/min. The spectrum was registered for freshly prepared apoLf and holoLf solutions in IF, as well as for supernatants containing protein released under simulated gastric and intestinal fluids. The collected spectra were further analyzed using the BeStSel method [50] to calculate the molar circular dichroism and estimate the composition of the secondary structure in percentage. The structure of diferric bovine Lf from PDB (1BLF [6]) was used as a reference. 

The ability of Lf released from MPs to rebind iron ions was tested via its incubation with Fe^3+^ in the presence of nitrilotriacetic acid (NTA) in a molar ratio of Lf:Fe:NTA 1:4:4 [47]. NTA was used as a weak chelator for Fe^3+^ ions to avoid nonspecific binding to the protein [47]. After 1 h of incubation at 37 °C, the samples were washed by centrifugation on AmiconUltra-0.5 mL 30 kDa filters to remove unbound Fe^3+^ ions, and the protein saturation level was determined as described in Section 2.1 and Section 2.4. 

## 3. Results and Discussion

### 3.1. Microparticles Synthesis and Characterization 

Empty MPs and MPs containing Lf saturated with Fe^3+^ ions (holoLf) were prepared from Eudragit^®^ RS following the procedure developed by Mendoza et al. [39] with some modifications: the internal aqueous phase was not acidified to avoid Fe^3+^ dissociation from holoLf, and sonication parameters were optimized with a QSonica 500 sonicator. Note that the encapsulation of bovine serum albumin (pI 4.7–5 [51]) was unsuccessful when neutral pH was used in the aqueous internal phase, whereas Lf (pI 8.5–9 [52]) was efficiently encapsulated at this pH. This originated from differences in the pI and in the net charge of the protein under given conditions. The obtained data suggest that the developed procedure can be useful for proteins with a positive charge. MPs were obtained with a good yield of (67 ± 1)% for empty MPs and (66 ± 5)% for holoLf-loaded MPs. The holoLf encapsulation efficiency (EE%) determined by the direct and indirect approaches reached a very high value of (94.4 ± 2.9)% and (90.6 ± 0.8)%, respectively, confirming a very good agreement between the applied methods. The loading capacity (LC%) reached the value of (9.7 ± 0.4)%. Equally high values of EE% and LC% have already been reported by Mendoza et al. for bovine serum albumin and horseradish peroxidase [39]. As mentioned in the introduction, such a high value for EE% outperforms other recently developed systems reporting EE% of 50–75% [36,37,38]. The high LC% value obtained is superior to the majority of the previously reported systems for Lf encapsulation and at a level similar to the innovative systems developed by Kanwar et al. (0.17 mg of protein per 1 mg of nanocapsules) [53] or Kiryukhin et al. (0.11 mg of protein per 1 mg of microparticles) [54]. 

**Figure 1:**
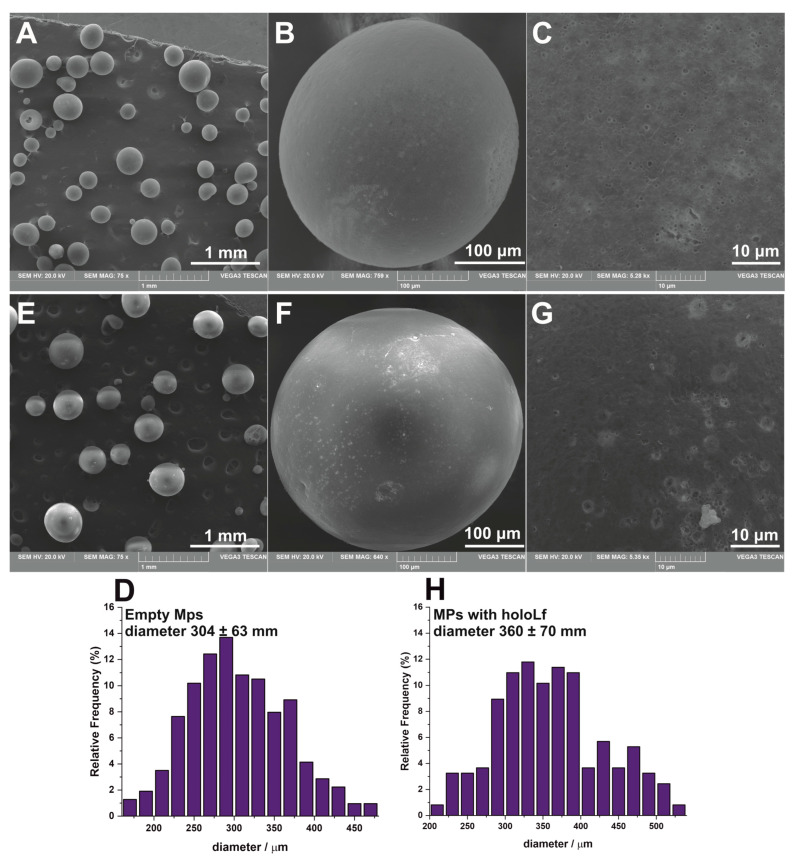
SEM images for empty (**A**–**C**) and holoLf-loaded (**E**–**G**) microparticles prepared from Eudragit^®^ RS followed by lyophilization shown with different magnifications (right panel shows the crack- and pinhole-free MP surfaces). (**D**) The size distribution histogram for empty MPs (based on 100 diameter measurements for each of the five independent samples) and (**H**) for MPs loaded with holoLf (based on 100 diameter measurements for each of the three independent samples).

The freeze-drying method is often applied for the preparation of pharmaceutical formulations to facilitate their storage, so the obtained MPs were lyophilized according to the protocol described in the experimental section. SEM images of gold-coated lyophilized MPs are presented in Figure 1. Both empty (SEM images in Figure 1A–C) and protein-loaded MPs (SEM images in Figure 1E–G) have a spherical morphology and lack of cracks or pores on their surfaces, which aid in the preservation of the loaded cargo under simulated gastric conditions. The protein-loaded MPs have a slightly larger diameter (360 ± 70 μm) than empty MPs (304 ± 63 μm). A detailed diameter distribution is presented in the histograms in Figure 1D,H. 

### 3.2. Protein Release from the MPs

**Figure 2:**
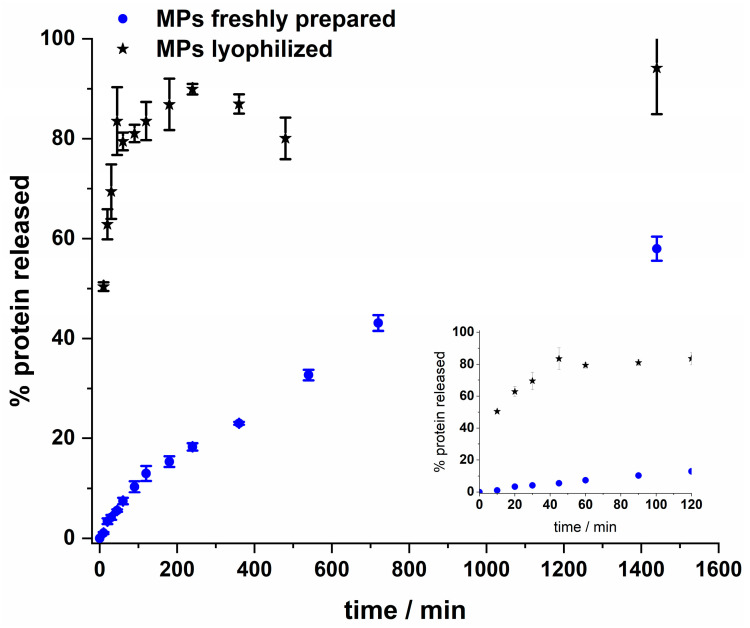
Lf release profile for lyophilized and freshly prepared MPs. MPs suspended in 10 times diluted PBS were placed under magnetic stirring at 300 rpm at room temperature. The released protein was quantified by the BCA protocol using the supernatant from the MPs. Insert: Release profile presented in the first 2 h.

The Lf release profile for freshly prepared and lyophilized MPs was performed in 10 times diluted PBS at room temperature (ca. 20 °C) and is presented in Figure 2. In the case of lyophilized MPs, a fast release was observed, and after 45–60 min, the entire cargo was released, and a similar profile was obtained at 37 °C. 

The release profile for freshly prepared MPs was sustained; less than 10% of the protein was released within the first hour. Although there are no visible cracks or pores in the SEM images of lyophilized MPs, the results from the release profile experiment suggest that their integrity/structure is affected by the freeze-drying method under applied conditions. Currently, a suitable method for drying MPs loaded with Lf for longer storage is under development. The obtained protein release data from freshly prepared MPs were further analyzed, and mathematical models were applied. First-order, Higuchi, Peppas–Sahlin, and Lindner–Lippold mathematical models were considered and the best fit (R^2^ = 0.994) was obtained for the Peppas–Sahlin model (Formula (1), Figure S3). According to the obtained values, the release is likely to have a mixed release mechanism, as suggested by the value of n = 0.60, with a predominance of the diffusion mechanism (k_1_ = 0.064) related to Fickian diffusion and a minor contribution of the relaxational mechanism (k_2_ = 0.0068) associated with the polymer matrix disintegration [55].

(1) $$f=k_{1}t^{n}+k_{2}t^{2n}$$

where f is a fraction of the released compound, k_1_ and k_2_ are constants related to Fickian diffusional contribution and relaxational contribution, respectively, n is a coefficient related to the type of the mechanism (for mixed mechanism 0.43 < n < 0.80 [55]), and t is the release time (h).

**Figure 3:**
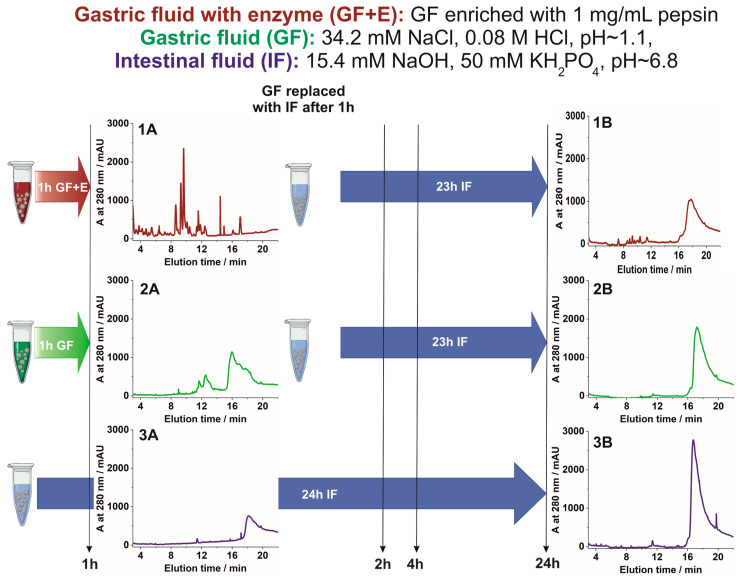
Scheme of the studies on the release of Lf from MPs performed in simulated gastric and intestinal fluids. (**1A**,**2A**,**3A**) are chromatograms obtained for supernatants collected from the MPs suspended in GF + E, GF, and IF, respectively, after 1 h of incubation. (**1B**,**2B**) are chromatograms obtained for supernatants collected from the MPs kept for 1 h in GF + E and GF, respectively, followed by 23 h of incubation in IF. (**3B**) is a chromatogram for a supernatant collected from the MPs kept for 24 h in IF.

Further studies on the release of Lf under gastric conditions were performed for freshly prepared MPs. The MPs were suspended in (i) gastric fluid enriched with pepsin (GF + E), (ii) gastric fluid without enzyme (GF), or (iii) intestinal fluid (IF) as schematically shown in Figure 3, together with the compositions of the fluids. After 1 h, the entire gastric juice was removed and collected for further analysis. The MPs were washed three times with IF and suspended in a fresh portion of IF to simulate release in the intestine. After another 1, 3, and 23 h, aliquots of the supernatant from the MPs were collected and analyzed for protein composition. MPs suspended only in IF aliquots of the supernatant were collected from the MPs after 1, 2, 4, and 24 h for comparison. To evaluate Lf digestion without MP protection, unprotected holoLf was added to GF + E at a final concentration of 10 mg/mL, and the sample was analyzed after 30 and 60 min.

The HPLC chromatogram obtained for the supernatant collected from holoLf-loaded MPs kept in GF + E solution for 1 h contained numerous sharp peaks at the elution time between 4 and 13 min (Figure 3, chromatogram 1A). The observed peaks, originated from short peptides, formed as a result of the pepsin-catalyzed proteolysis of Lf. A similar chromatographic peak pattern was observed for unprotected Lf incubated with GF + E (Figure S4). Despite the high protein concentration, it was completely digested after 30 min, and the peak assigned to the intact protein molecule was not observed at all. The complete digestion of Lf is consistent with numerous in vitro studies showing that unbound and unprotected Lf undergoes significant proteolysis. The percentage of structurally intact or undigested forms of Lf exposed to simulated gastric fluid was marginal as previously reported [28,56,57]. Only one peak was present in the chromatogram obtained for the supernatant collected after removing the Lf-loaded MPs from GF + E and placing them in IF for 23 h (Figure 3, chromatogram 1B). This indicates that a significant amount of Lf was protected from the simulated proteolytic conditions and released into the intestine-simulated environment. Lf released from MPs kept in pepsin-depleted GF for 1 h also underwent some structural changes, and the distorted peak was observed for Lf (Figure 3, chromatogram 2A). Changes in the elution time and shape of the peak suggest the partial unfolding of Lf, as it is usually observed when the protein is exposed to a low pH [58]. Lf was readily and gradually released from MPs in IF, and the chromatograms obtained for the collected supernatants suggest that the protein was intact (Figure 3 chromatograms 1B, 2B, 3A, and 3B, Figures S5–S7). 

**Figure 4:**
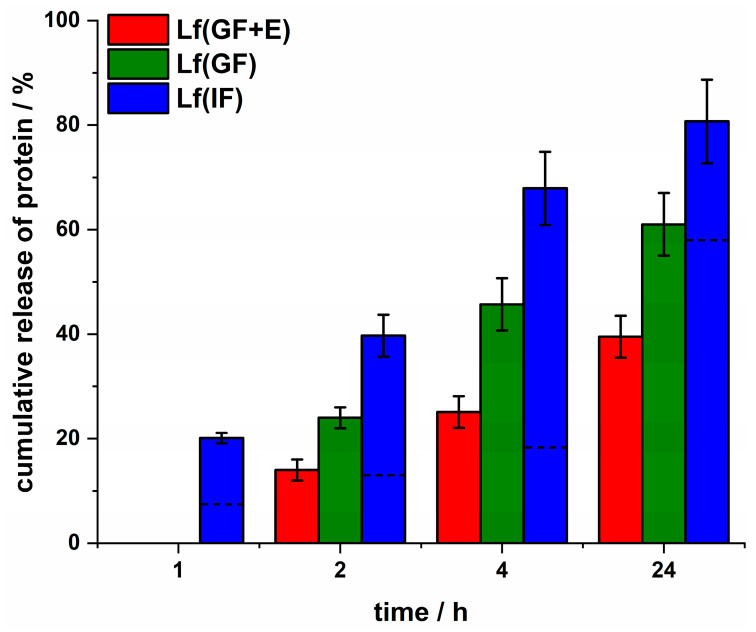
The Lf cumulative release under simulated gastric conditions. The percentage of the protein released from MPs was calculated as the ratio between the peak area of the protein in the HPLC separation and the peak area obtained for the sonicated sample in which the whole present protein was released. Incubation conditions: Lf(GF + E) MPs kept 1 h in GF + E followed by incubation in IF (red bars), Lf(GF) MPs kept 1 h in GF followed by incubation in IF, Lf(IF) MPs kept in IF. Lf (IF) bars with a dotted line present % of the protein released from the MPs in PBS taken from Figure 2 for comparison.

To obtain the release characteristics under various gastric conditions, all collected supernatants after 1, 2, 4, and 24 h were subjected to HPLC separation (Figures S3–S5), and the peak area of intact Lf was analyzed. MPs remaining after the experiment were sonicated (pulse sonication, 10 cycles of 10 s at 40% amplitude) to disintegrate MPs and release their entire cargo (Figures S5–S7). The area of the peak obtained for the supernatant collected for MPs incubated for 24 h in IF followed by sonication was related to 100% of the protein that could be released from the prepared MPs. The area of all other peaks was divided by it to normalize the results. The release of protein under various simulated gastric conditions is presented in Figure 4. The recovery of Lf from MPs incubated only in IF for 24 h was ca. 80%, whereas when MPs were pre-incubated for 1 h in GF + E, it was ca. 40%. It is a good result considering that free Lf is entirely digested under similar conditions (Figure S4). Therefore, the encapsulation of Lf in MPs protects the protein against proteolysis. Generally, regardless of the applied conditions, the protein is gradually released, and the rate of release is not influenced by the pre-incubation conditions; it is related to the pH-independent swelling of the polymer and the degradation of the MPs. It must be noted that not only Lf released into GF + E is degraded. As the result of sustained erosion of the MPs, pepsin, which is a smaller protein than Lf, may penetrate inside the outer layer of the particle and digest the entrapped protein, which overall leads to a higher loss of protein than expected from the release studies. When MPs are placed in IF, the higher pH inactivates pepsin, so no further degradation of Lf is observed. The release profile obtained in PBS (Figure 2) differs from the release characteristics obtained in simulated gastric and intestinal fluids (Figure 4). Lf is readily released and to a higher level in simulated gastric fluids, so using PBS in the studies might provide different outcomes and cannot be directly related to gastric conditions. 

### 3.3. Characterization of Lf Released from MPs

#### 3.3.1. Iron Saturation Level 

The collected supernatants were divided into two parts. The first was mineralized and analyzed by ICP-MS to determine the ionic iron content. The second was analyzed for Lf concentration using the BCA protocol. The iron saturation of the protein was calculated for all samples released from the MPs. The Lf released from the MPs pre-incubated in GF + E or GF for 1 h followed by incubation in IF was saturated with iron ions at (33 ± 5)%, and the saturation level remained constant regardless of the time of supernatant collection. Considering that in GF, the free holoLf (iron saturation of 98.3%) releases iron ions completely within one minute (Figure S8), the preservation of one-third of the iron saturation levels of Lf encapsulated in MPs under such conditions is a great result. Lf released from MPs incubated in IF remained saturated in ca. 92%, losing a negligible amount of bound iron ions within 24 h. Free holoLf at pH ca. 6.8 released iron ions only at a minor degree (Figure S8), which makes it a very good iron scavenger under intestinal conditions. Additionally, it proves that the encapsulation process does not cause the dissociation of iron ions, so actually holoLf is trapped in the MPs. The obtained results suggest that MPs can deliver intact Lf to the intestinal tract, and its iron saturation level is twice as high as that typically found in native Lf, whose saturation is ca. 15–20% [45,47]. It should be noted that the simulated gastric fluid model used in this study, i.e., pH~1.1 and 1 h of incubation, is very harsh. There is a large discrepancy in the models used in the literature as was mentioned in the introduction. In some protocols, higher pH, even up to 4.0 [30], is used to simulate the stomach conditions; thus, by using these protocols, even higher saturation levels would be expected.

#### 3.3.2. Structural Stability and Iron Scavenging Ability

**Figure 5:**
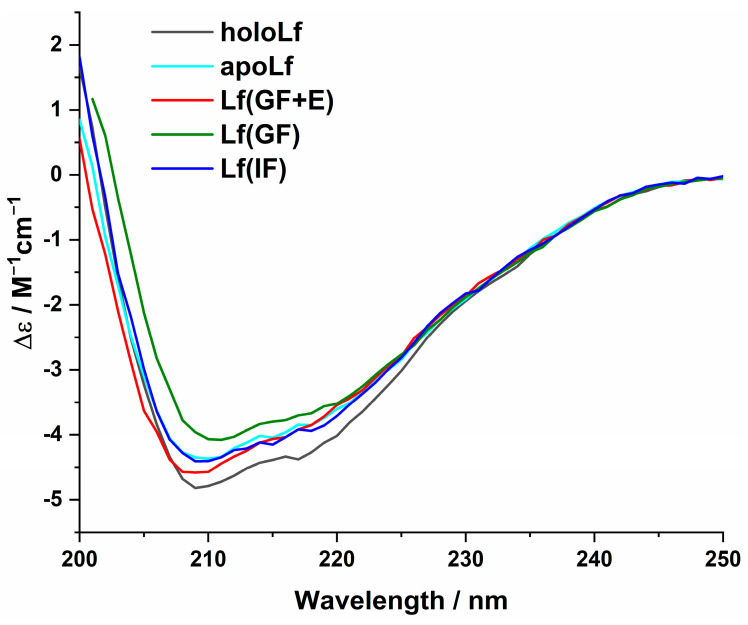
CD spectra of holoLf and apoLf as well as Lf released from MPs kept 1 h in GF + E followed by 23 h of incubation in IF–Lf(GF + E), kept 1 h in GF followed by 23 h of incubation in IF–Lf(GF), and kept 24 h in IF–Lf(IF). Spectra correspond to the sample analyzed using HPLC from Figure 3: chromatograms 1B, 2B, and 3B.

**Figure 6:**
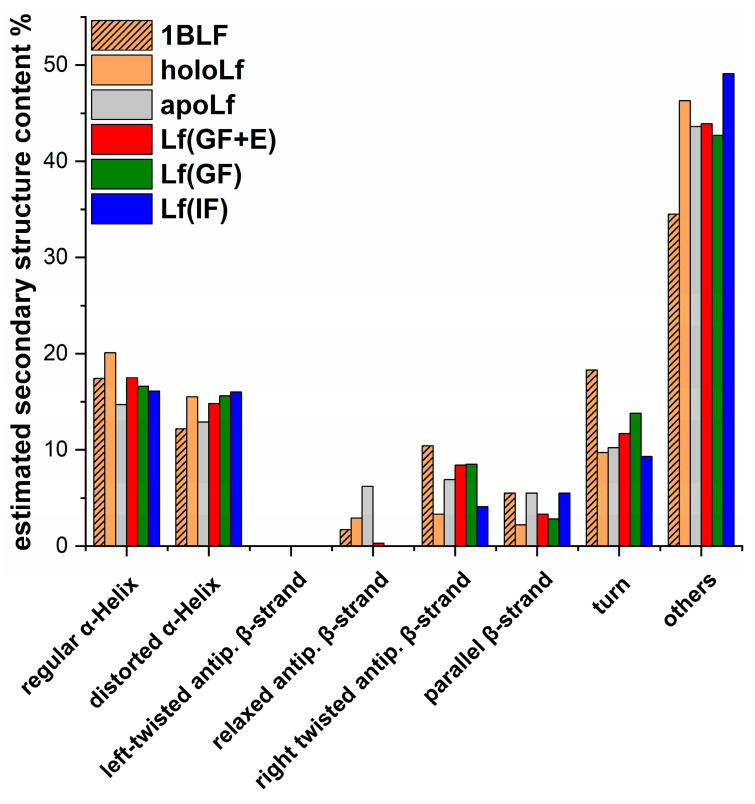
Calculated secondary structure composition in percentage (%) for PDB structure of diferric bovine Lf 1BLF [6], holoLf, apoLf, and Lf released from MPs kept 1 h in GF + E followed by 23 h of incubation in IF–Lf(GF + E), kept 1 h in GF followed by 23 h of incubation in IF–Lf(GF), and kept 24 h in IF–Lf(IF). Estimation was carried out using the BeStSel method [50]. Structure estimations correspond to the sample analyzed using HPLC from Figure 3: chromatograms 1B, 2B, and 3B.

The stability of the protein released from MPs under simulated gastric conditions was monitored by circular dichroism. The obtained CD spectra are presented in Figure 5. Based on the collected CD spectra, the secondary structure composition was calculated using the BeStSel method [50], and the obtained results are presented in Figure 6. Furthermore, the secondary structure composition taken from the PDB file for diferric bovine Lf (PDB ID: 1BLF [6]) was presented for comparison. The lack of meaningful differences in the shape of the CD spectrum for apoLf or holoLf and Lf released from MPs after 24 h demonstrates the protein preserved its secondary structure. This preservation was observed regardless of the applied conditions, indicating that the protein was protected by MPs exposed to simulated gastric fluid containing proteolytic enzymes. Estimations carried out by applying the BeStSel method [50] support this finding. Only minor differences in the secondary structure composition are visible between the references (holoLf and apoLf) and the Lf released from MPs under gastric conditions. The difference between all estimations based on experimental data and estimations based on the PDB file is more evident, especially the content of other structures (among others: bends, loops, and irregular regions), which is higher for all the estimations based on experimental data. It should be noted that an estimation based on PDB crystal structure naturally has a higher content of ordered structures than those on data collected for protein incubated in aqueous solutions for 24 h. Furthermore, a protein released from MPs exposed to GF or GF + E for which iron saturation drops down to ca. (33 ± 5)% retained the ability to rebind iron ions. The released protein was able to scavenge iron ions very efficiently, reaching full saturation ((100 ± 5)%) after exposure to a solution of nitrilotriacetic acid (NTA)-Fe^3+^ at a molar concentration ratio of Lf:Fe:NTA = 1:4:4 for 1 h, conditions that are typically applied to obtain holoLf [47]. 

## 4. Conclusions

HoloLf was successfully encapsulated into MPs formed using Eudragit^®^ RS with a very high encapsulation efficiency of over 90% and a loading capacity of ca. 0.10 mg of protein per 1 mg of MPs and iron content ca. 92%. A suitable morphology of the obtained MPs provided sustained release of Lf under simulated gastric conditions. Pre-incubation of Lf-loaded MPs under conditions that simulate the stomach led to only partial protein proteolysis and iron ions release. The protective role of MPs was demonstrated by the release of the remaining protein under conditions that simulated the lower part of the gastric tract with an efficiency of ca. 40% compared to the originally loaded amount of protein. The iron saturation level of the released Lf was ca. 33%, which is expected to be beneficial given previous reports in the literature on synergistic effects of holoLf with anticancer or antimicrobial drugs [11]. Notably, the released Lf was structurally intact and its ability to readily rebind iron ions was preserved.

The pH of gastric fluids strongly depends on the buffering capacity of food. In the case of higher pH and shorter exposure time related to the increased emptying time of the stomach, an even better release profile would be expected. The lower loss of the protein due to the shorter time of proteolysis and a reduced amount of iron ions released at higher pH should be observed. Thus, the obtained MPs loaded with Lf could be a promising material for further studies on the co-encapsulation of Lf and active agents against intestinal/colorectal diseases.

## Data Availability

The data presented in this study are available in the main text and Supplementary Materials.

## Supplementary Materials

The following supporting information can be downloaded at: https://www.mdpi.com/article/10.3390/nano13182524/s1, Figure S1: HPLC chromatograms for Lf standard solutions; Figure S2: HPLC calibration method for holoLf; Figure S3: Lf release profile applying the Peppas–Sahlin kinetic release profile; Figure S4: Chromatograms obtained for unprotected holoLf proteolysis; Figure S5: Chromatograms obtained for supernatants collected from the MPs kept 1 h in GF + E followed by 23 h of incubation in IF; Figure S6: Chromatograms obtained for supernatants collected from the MPs kept 1 h in GF followed by 23 h of incubation in IF; Figure S7: Chromatograms obtained for supernatants collected from the MPs kept 24 h in IF; Figure S8: Kinetics of iron released from holoLf depending on the pH of the simulated fluid.

## References

[1] Moreno-Expósito L., Illescas-Montes R., Melguizo-Rodríguez L., Ruiz C., Ramos-Torrecillas J., de Luna-Bertos E. (2018). Multifunctional capacity and therapeutic potential of lactoferrin. Life Sci..

[2] Kowalczyk P., Kaczyńska K., Kleczkowska P., Bukowska-Ośko I., Kramkowski K., Sulejczak D. (2022). The Lactoferrin Phenomenon—A Miracle Molecule. Molecules.

[3] Drago-Serrano M.E., Campos-Rodríguez R., Carrero J.C., Delagarza M. (2017). Lactoferrin: Balancing ups and downs of inflammation due to microbial infections. Int. J. Mol. Sci..

[4] Rosa L., Cutone A., Lepanto M., Paesano R., Valenti P. (2017). Lactoferrin: A Natural Glycoprotein Involved in Iron and Inflammatory Homeostasis. Int. J. Mol. Sci..

[5] Ashraf M.F., Zubair D., Bashir M.N., Alagawany M., Ahmed S., Shah Q.A., Buzdar J.A., Arain M.A. (2023). Nutraceutical and Health-Promoting Potential of Lactoferrin, an Iron-Binding Protein in Human and Animal: Current Knowledge. Biol. Trace Elem. Res..

[6] Moore S.A., Anderson B.F., Groom C.R., Haridas M., Baker E.N. (1997). Three-dimensional structure of diferric bovine lactoferrin at 2.8 Å resolution. J. Mol. Biol..

[7] Li Y., Dong L., Mu Z., Liu L., Yang J., Wu Z., Pan D., Liu L. (2022). Research Advances of Lactoferrin in Electrostatic Spinning, Nano Self-Assembly, and Immune and Gut Microbiota Regulation. J. Agric. Food Chem..

[8] Aisen P., Leibman A. (1972). Lactoferrin and transferrin: A comparative study. BBA—Protein Struct..

[9] Gruden Š., Poklar Ulrih N. (2021). Diverse Mechanisms of Antimicrobial Activities of Lactoferrins, Lactoferricins, and Other Lactoferrin-Derived Peptides. Int. J. Mol. Sci..

[10] Jenssen H., Hancock R. (2009). Antimicrobial properties of lactoferrin. Biochimie.

[11] Gajda-Morszewski P., Brindell M. (2021). Lactoferrin as a Potent Natural Supplement Exhibiting a Synergistic Effect with Drugs in Antimicrobial and Anticancer Therapies. Curr. Protein Pept. Sci..

[12] Diarra M.S., Petitclerc D., Lacasse P. (2002). Effect of lactoferrin in combination with penicillin on the morphology and the physiology of *Staphylococcus aureus* isolated from bovine mastitis. J. Dairy Sci..

[13] Marshall L.J., Oguejiofor W., Price R., Shur J. (2016). Investigation of the enhanced antimicrobial activity of combination dry powder inhaler formulations of lactoferrin. Int. J. Pharm..

[14] Kanwar J.R., Palmano K.P., Sun X., Kanwar R.K., Gupta R., Haggarty N., Rowan A., Ram S., Krissansen G.W. (2008). “Iron-saturated” lactoferrin is a potent natural adjuvant for augmenting cancer chemotherapy. Immunol. Cell Biol..

[15] Sun X., Jiang R., Przepiorski A., Reddy S., Palmano K.P., Krissansen G.W. (2012). “Iron-saturated” bovine lactoferrin improves the chemotherapeutic effects of tamoxifen in the treatment of basal-like breast cancer in mice. BMC Cancer.

[16] Bellés A., Aguirre-Ramírez D., Abad I., Parras-Moltó M., Sánchez L., Grasa L. (2022). Lactoferrin modulates gut microbiota and Toll-like receptors (TLRs) in mice with dysbiosis induced by antibiotics. Food Funct..

[17] Wang A., Duncan S.E., Lesser G.J., Ray W.K., Dietrich A.M. (2018). Effect of lactoferrin on taste and smell abnormalities induced by chemotherapy: A proteome analysis. Food Funct..

[18] Moastafa T.M., El-Sissy A.E.-D.E., El-Saeed G.K., Koura M.S.E.-D. (2014). Study on the Therapeutic Benefit on Lactoferrin in Patients with Colorectal Cancer Receiving Chemotherapy. Int. Sch. Res. Notices.

[19] European Commission (2012). European Commission Implementing Decision No. 2012/727/EU Authorizing the Placing on the Market of Bovine Lactoferrin as a Novel Food Ingredient under Regulation (EC) No. 258/97 of the European Parliament and of the Council.

[20] Wang B., Timilsena Y.P., Blanch E., Adhikari B. (2019). Lactoferrin: Structure, function, denaturation and digestion. Crit. Rev. Food Sci. Nutr..

[21] Yao X., Bunt C., Cornish J., Quek S.-Y., Wen J. (2014). Stability of Bovine Lactoferrin in Luminal Extracts and Mucosal Homogenates from Rat Intestine: A Prelude to Oral Absorption. Chem. Biol. Drug Des..

[22] Wang B., Timilsena Y.P., Blanch E., Adhikari B. (2017). Mild thermal treatment and in-vitro digestion of three forms of bovine lactoferrin: Effects on functional properties. Int. Dairy J..

[23] Shimoni G., Shani Levi C., Levi Tal S., Lesmes U. (2013). Emulsions stabilization by lactoferrin nano-particles under in vitro digestion conditions. Food Hydrocoll..

[24] David-Birman T., Mackie A., Lesmes U. (2013). Impact of dietary fibers on the properties and proteolytic digestibility of lactoferrin nano-particles. Food Hydrocoll..

[25] Grosvenor A.J., Haigh B.J., Dyer J.M. (2014). Digestion proteomics: Tracking lactoferrin truncation and peptide release during simulated gastric digestion. Food Funct..

[26] Troost F.J., Saris W.H.M., Brummer R.-J.M. (2002). Orally Ingested Human Lactoferrin Is Digested and Secreted in the Upper Gastrointestinal Tract In Vivo in Women with Ileostomies. J. Nutr..

[27] Kuwata H., Yamauchi K., Teraguchi S., Ushida Y., Shimokawa Y., Toida T., Hayasawa H. (2001). Functional Fragments of Ingested Lactoferrin Are Resistant to Proteolytic Degradation in the Gastrointestinal Tract of Adult Rats. J. Nutr..

[28] Furlund C.B., Ulleberg E.K., Devold T.G., Flengsrud R., Jacobsen M., Sekse C., Holm H., Vegarud G.E. (2013). Identification of lactoferrin peptides generated by digestion with human gastrointestinal enzymes. J. Dairy Sci..

[29] Fan F., Shi P., Chen H., Tu M., Wang Z., Lu W., Du M. (2019). Identification and availability of peptides from lactoferrin in the gastrointestinal tract of mice. Food Funct..

[30] Troost F.J., Steijns J., Saris W.H.M., Brummer R.-J.M. (2001). Gastric Digestion of Bovine Lactoferrin In Vivo in Adults. J. Nutr..

[31] Gleeson J.P., Fein K.C., Chaudhary N., Doerfler R., Newby A.N., Whitehead K.A. (2021). The enhanced intestinal permeability of infant mice enables oral protein and macromolecular absorption without delivery technology. Int. J. Pharm..

[32] Dupont T.L. (2019). Donor Milk Compared with Mother’s Own Milk. Hematology, Immunology and Genetics.

[33] Abdallah F.B., el Hage Chahine J.-M. (2000). Transferrins: Iron release from lactoferrin. J. Mol. Biol..

[34] Ong R., Cornish J., Wen J. (2022). Nanoparticular and other carriers to deliver lactoferrin for antimicrobial, antibiofilm and bone-regenerating effects: A review. BioMetals.

[35] Abad I., Conesa C., Sánchez L. (2021). Development of Encapsulation Strategies and Composite Edible Films to Maintain Lactoferrin Bioactivity: A Review. Materials.

[36] Guan R., Ma J., Wu Y., Lu F., Xiao C., Jiang H., Kang T. (2012). Development and characterization of lactoferrin nanoliposome: Cellular uptake and stability. Nanoscale Res. Lett..

[37] Ono K., Sakai H., Tokunaga S., Sharmin T., Aida T.M., Mishima K. (2020). Encapsulation of Lactoferrin for Sustained Release Using Particles from Gas-Saturated Solutions. Processes.

[38] Varela-Fernández R., García-Otero X., Díaz-Tomé V., Regueiro U., López-López M., González-Barcia M., Isabel Lema M., Javier Otero-Espinar F. (2022). Lactoferrin-loaded nanostructured lipid carriers (NLCs) as a new formulation for optimized ocular drug delivery. Eur. J. Pharm. Biopharm..

[39] Gracia R., Yus C., Abian O., Mendoza G., Irusta S., Sebastian V., Andreu V., Arruebo M. (2018). Enzyme structure and function protection from gastrointestinal degradation using enteric coatings. Int. J. Biol. Macromol..

[40] Food and Drug Administration (2017). GRAS Notice (GRN) No. 710.

[41] European Commission (2011). European Commission Regulation No. 1129/2011 amending Annex II to Regulation (EC) No. 1333/2008 of the European Parliament and of the Council by Establishing a Union List of Food Additives.

[42] Patra C.h.N., Priya R., Swain S., Kumar Jena G., Panigrahi K.C., Ghose D. (2017). Pharmaceutical significance of Eudragit: A review. Futur. J. Pharm. Sci..

[43] Tarcha P. (1991). Polymers for Controlled Drug Delivery.

[44] Eisele J., Haynes G., Rosamilia T. (2011). Characterisation and toxicological behaviour of Basic Methacrylate Copolymer for GRAS evaluation. Regul. Toxicol. Pharmacol..

[45] Gajda-Morszewski P., Śpiewak-Wojtyła K., Oszajca M., Brindell M. (2019). Strategies for oral delivery of metal-saturated lactoferrin. Curr. Protein Pept. Sci..

[46] Kanwar J.R., Mahidhara G., Kanwar R.K. (2012). Novel alginate-enclosed chitosan-calcium phosphate-loaded iron-saturated bovine lactoferrin nanocarriers for oral delivery in colon cancer therapy. Nanomedicine.

[47] Majka G., Spiewak K., Kurpiewska K., Heczko P., Stochel G., Strus M., Brindell M., Śpiewak K., Kurpiewska K., Heczko P. (2013). A high-throughput method for the quantification of iron saturation in lactoferrin preparations. Anal. Bioanal. Chem..

[48] Rasband W.S. (1997–2015) ImageJ. National Institutes of Health, Bethesda, Maryland, USA. http://imagej.nih.gov/ij.

[49] Schneider C.A., Rasband W.S., Eliceiri K.W. (2012). NIH Image to ImageJ: 25 years of image analysis. Nat. Methods.

[50] Micsonai A., Wien F., Kernya L., Lee Y.-H., Goto Y., Réfrégiers M., Kardos J. (2015). Accurate secondary structure prediction and fold recognition for circular dichroism spectroscopy. Proc. Natl. Acad. Sci. USA.

[51] Wïniewska M., Szewczuk-Karpisz K., Sternik D. (2015). Adsorption and thermal properties of the bovine serum albumin-silicon dioxide system. J. Therm. Anal. Calorim..

[52] Brock J.H. (1997). Lactoferrin Structure-Function Relationships. Lactoferrin.

[53] Mahidhara G., Kanwar R.K., Roy K., Kanwar J.R. (2015). Oral administration of iron-saturated bovine lactoferrin–loaded ceramic nanocapsules for breast cancer therapy and influence on iron and calcium metabolism. Int. J. Nanomed..

[54] Kiryukhin M.V., Lim S.H., Lau H.H., Antipina M., Khin Y.W., Chia C.Y., Harris P., Weeks M., Berry C., Hurford D. (2021). Surface-reacted calcium carbonate microparticles as templates for lactoferrin encapsulation. J. Colloid Interface Sci..

[55] Peppas N.A., Sahlin J.J. (1989). A simple equation for the description of solute release. III. Coupling of diffusion and relaxation. Int. J. Pharm..

[56] Eriksen E.K., Holm H., Jensen E., Aaboe R., Devold T.G., Jacobsen M., Vegarud G.E. (2010). Different digestion of caprine whey proteins by human and porcine gastrointestinal enzymes. Br. J. Nutr..

[57] Inglingstad R.A., Devold T.G., Eriksen E.K., Holm H., Jacobsen M., Liland K.H., Rukke E.O., Vegarud G.E. (2010). Comparison of the digestion of caseins and whey proteins in equine, bovine, caprine and human milks by human gastrointestinal enzymes. Dairy Sci. Technol..

[58] Mata L., Sánchez L., Headon D.R., Calvo M. (1998). Thermal Denaturation of Human Lactoferrin and Its Effect on the Ability to Bind Iron. J. Agric. Food Chem..

