# Supplementary material for: Encapsulation of Iron-Saturated Lactoferrin for Proteolysis Protection with Preserving Iron Coordination and Sustained Release
Source: Nanomaterials (Basel). 2023 Sep 8;13(18):2524. doi: 10.3390/nano13182524 (PMC10535829; doi:10.3390/nano13182524)
Supplement: Supplementary file 1 [file nanomaterials-13-02524-s001.zip › nanomaterials-2536599-supplementary.pdf]

Encapsulation of iron-saturated lactoferrin for proteolysis protection with preserving iron coordination and sustained release

Przemysław Gajda-Morszewski[1,2], Anna Poznańska[1], Cristina Yus[3,4], Manuel Arruebo[3,4,*] and Małgorzata Brindell[1,*]

1 Faculty of Chemistry, Jagiellonian University, Gronostajowa 2, 30-387 Krakow, Poland; pgmorszewski@doctoral.uj.edu.pl (P.G-M.), anna4.poznanska@student.uj.edu.pl (A.P.)

2 Jagiellonian University, Doctoral School of Exact and Natural Sciences, Prof. St. Łojasiewicza St 11, 30-348, Kraków, Poland

3 Instituto de Nanociencia y Materiales de Aragón (INMA), CSIC-Universidad de Zaragoza, Zaragoza 50009, Spain; cyargon@gmail.com (C.Y.)

4 Department of Chemical Engineering, University of Zaragoza, Campus Río Ebro-Edificio I+D, C/ Poeta Mariano Esquillor S/N, 50018 Zaragoza, Spain

*Correspondence: arruebom@unizar.es (M.A.); malgorzata.brindell@uj.edu.pl (M.B.)

Supplementary Material Section

Study of Interference Between Microparticle System Components and BCA Assay

Polymers (and especially hydrophobic ones) often interact with the dyes used for protein determination. To exclude the risk of such interference, empty MPs were prepared. A known amount of lactoferrin was added to the MPs to achieve concentrations of 0.5 and 1.0 mg/ml. Further samples were incubated together for 1 h at room temperature, sonicated until MPs disintegrated (pulse sonication, 10 cycles of 10 seconds at 40% amplitude), and centrifuged (15 min 14000 g). Lf concentration was measured by BCA assay, and recoveries were calculated for lower and higher Lf concentrations, respectively: (109±7)% and (95±5)%. The obtained recovery values, considering the random error, are close to 100% and suggest that the presence of polymers does not interfere with BCA assay. Additionally, protein concentrations determined by BCA were verified by HPLC using calibration curve presented below.

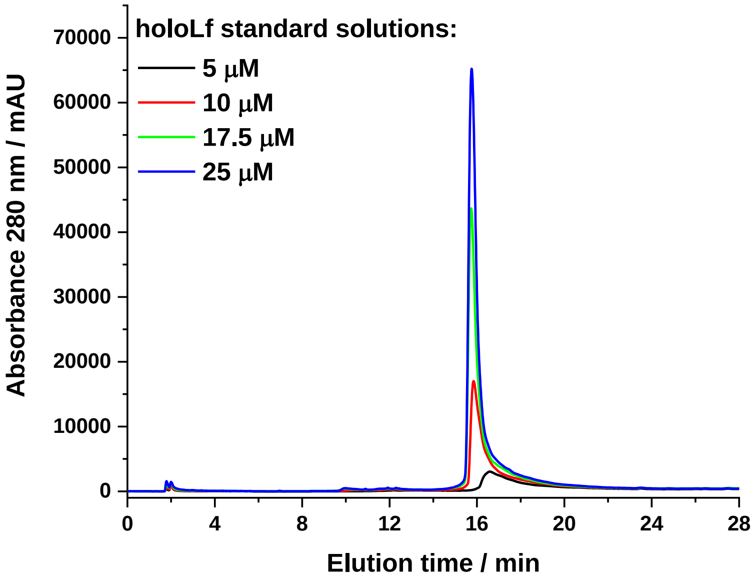

Figure S1. – Chromatograms for holoLf standard solutions containing 5; 10; 17.5; 25 μM of protein. Separation was performed on a Brownlee BioC18 column (150 × 4.6 mm; particle size 5 μm). Eluent

A: 0.05% TFA in water; eluent B: 0.05% TFA in acetonitrile. Gradient from 20% B to 70% B over 30 min with a flow rate of 1 ml/min at 30 °C. The detection was at 280 nm and the injection volume was 20 µl.

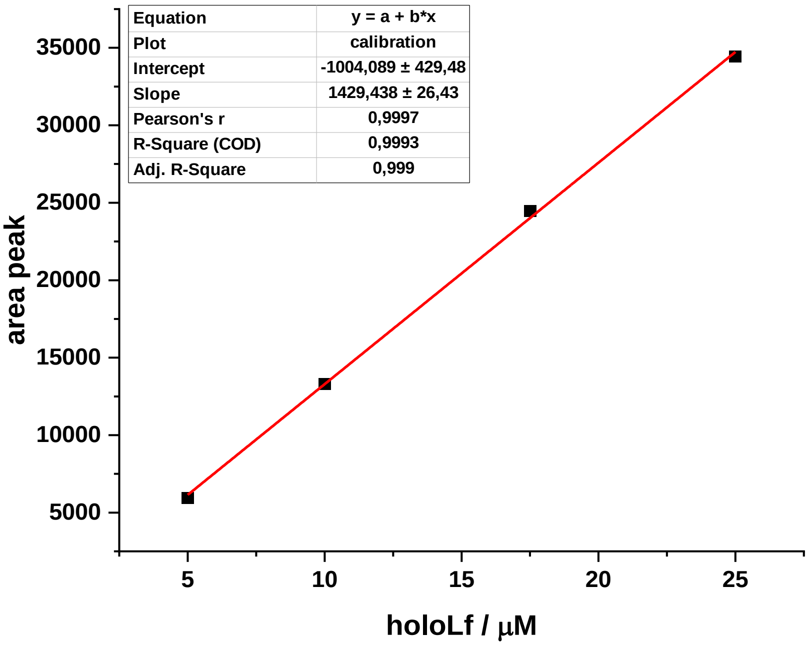

Figure S2. – Calibration curve of holoLf by the HPLC method. Calibration was performed for solutions containing 5; 10; 17.5; 25 µM of holoLf. Peaks presented on Figure S1 were integrated in OriginPro 9.7 software.

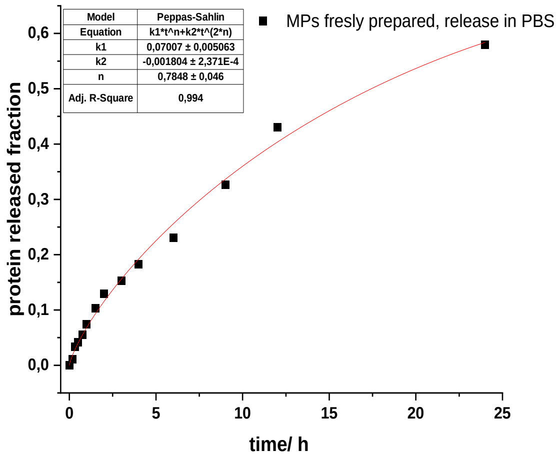

Figure S3. – Lf release profile for freshly prepared MPs suspended in 10 times diluted PBS. Experiment was carried out under magnetic stirring at 300 rpm at room temperature. The released protein was quantified by the BCA protocol using the supernatant from the MPs. Peppas-Sahlin kinetic model for cargo release was applied to the data.

Chromatograms obtained for samples collected during the release experiment in simulated fluids. The simulated fluids were prepared according to the 10th edition of the European Pharmacopeia: simulated gastric fluid without enzymes (**GF**, 34.2 mM NaCl, 0.08 M HCl, pH~1.1) or with a proteolytic enzyme (**GF+E**: **GF** enriched with 1 mg/ml pepsin; P7125, Sigma-Aldrich, >400 units/mg protein), and simulated intestinal fluid (**IF**, 15.4 mM NaOH, 50 mM KH_2PO_4, pH~6.8). Separation parameters are the same as denoted in caption for Figure S1.

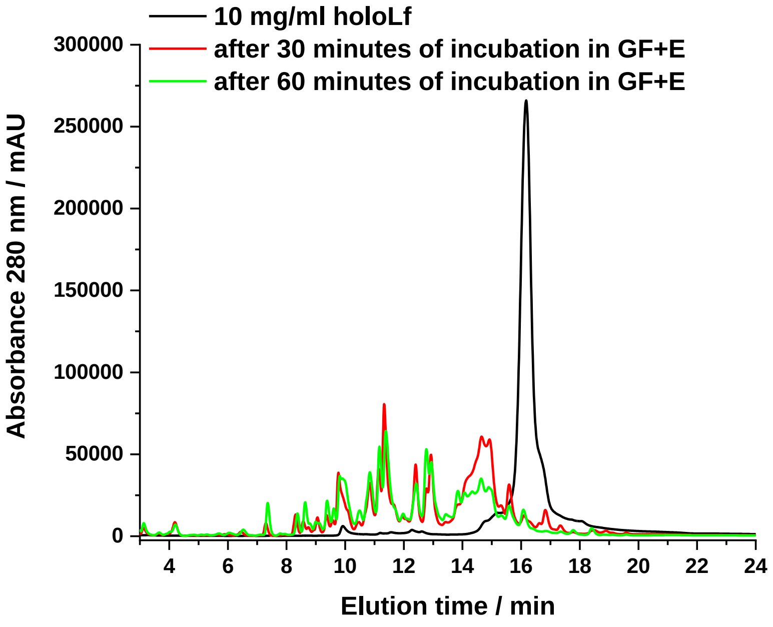

Figure S4. – Chromatograms obtained for unprotected holoLf (10 mg/ml) incubated with GF+E for 30 and 60 minutes at room temperature.

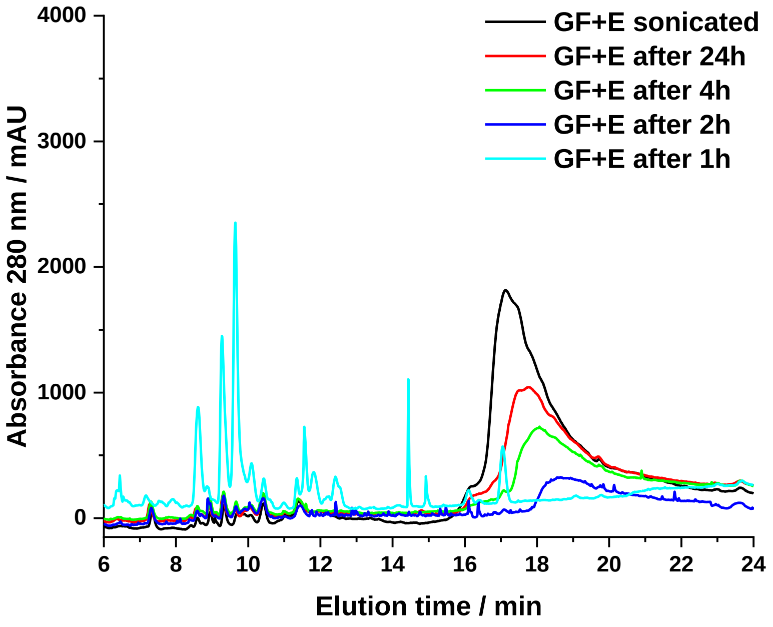

Figure S5. - Chromatograms obtained for supernatants collected from MPs kept 1 h in GF+E. After 1h all gastric fluid was collected for HPLC analysis and replaced with IF for another 23 h of incubation in IF. Samples of IF over MPs were taken for HPLC analysis after 2, 4, and 24 hours since the beginning of the experiment. Remaining MPs in IF after 24 h were sonicated until complete disintegration (pulse sonication, 10 cycles of 10 seconds at 40% amplitude), centrifuged (15 min 14 000 g), and supernatant was analyzed by HPLC (GF+E sonicated).

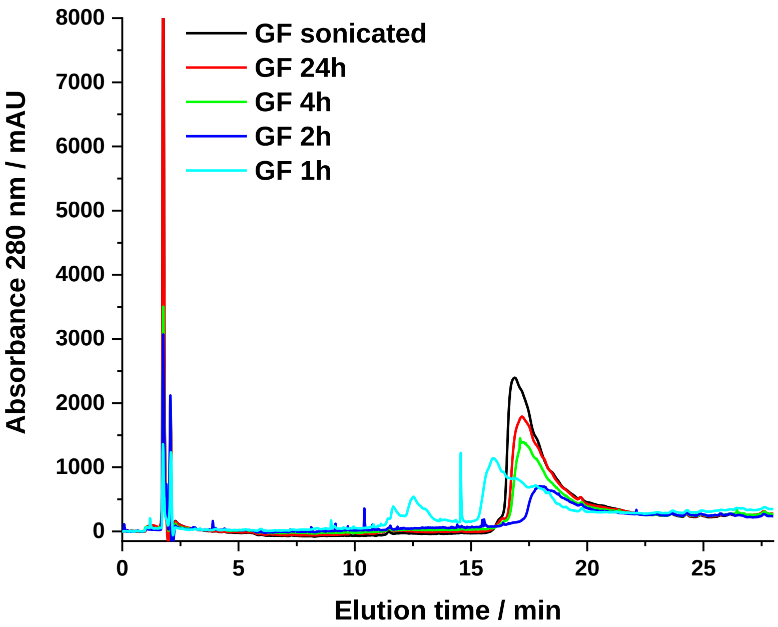

Figure S6. - Chromatograms obtained for supernatants collected from MPs kept 1 h in GF. After 1h all gastric fluid was collected for HPLC analysis and replaced with IF for another 23 h of incubation in IF. Samples of IF over MPs were taken for HPLC analysis after 2, 4, and 24 hours since the beginning of the experiment. Remaining MPs in IF after 24h were sonicated until complete disintegration (pulse sonication, 10 cycles of 10 seconds at 40% amplitude), centrifuged (15 min 14 000 g), and supernatant was analyzed by HPLC (GE sonicated).

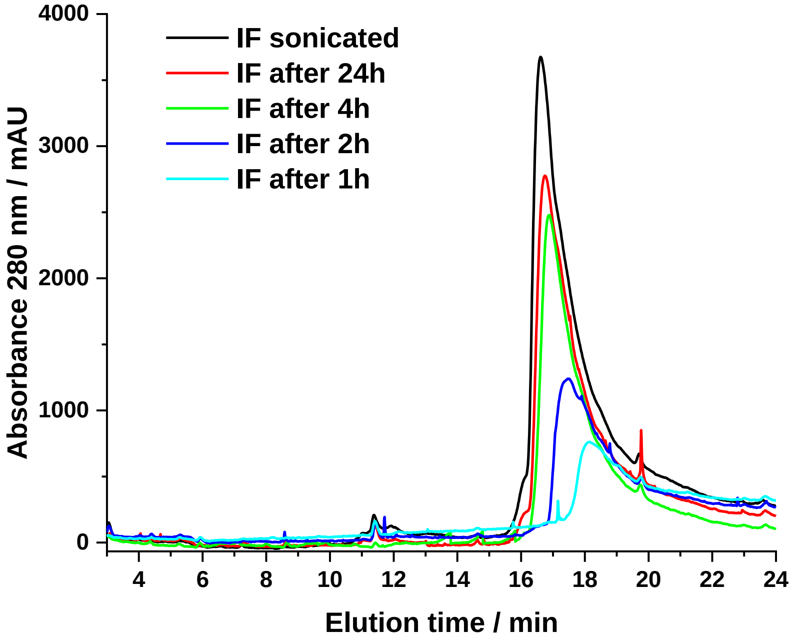

Figure S7. – Chromatograms obtained for supernatants collected from MPs kept 24 h in IF. Samples of IF from MPs were taken after 1, 2, 4, 24 hours since the beginning of the experiment for HPLC analysis. Remaining MPs in IF after 24h were sonicated until complete disintegration (pulse sonication, 10 cycles of 10 seconds at 40% amplitude), centrifuged (15 min 14000 g), and supernatant was analyzed by HPLC (IF sonicated).

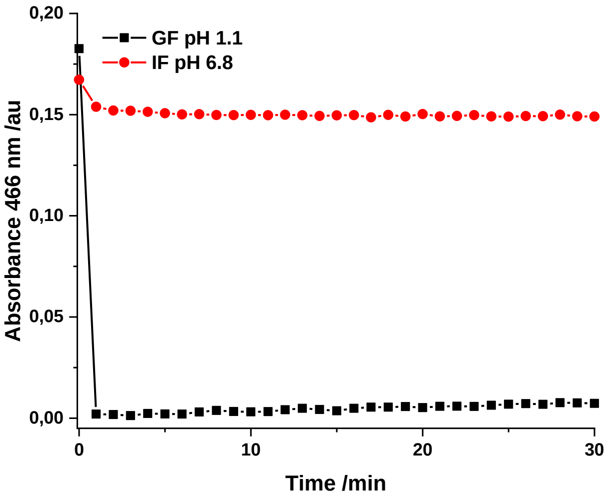

Figure S8. Kinetics of iron release from holoLf depending on the pH of the simulated fluid used. **GF** – gastric fluid containing 34.2 mM NaCl, 0.08 M HCl, pH~1.1 and **IF** – intestinal fluid containing 15.4 mM NaOH, 50 mM KH_2PO_4, pH~6.8. Absorbance was monitored at 466 nm, the band characteristic for holoLf. Reaction conditions: [holoLf] = 5 mg/ml, spectra recorded on PerkinElmer PDA UV/Vis Lambda 265 in a quartz tandem cuvette with a path length of 1.00 cm. Measurements were performed at 37 ^{o}C over 30 min with 1 min data interval.
